# Evaluation of usage-induced degradation of different endodontic file systems

**DOI:** 10.1038/s41598-021-88570-4

**Published:** 2021-04-27

**Authors:** Shekhar Bhatia, Venkateshbabu Nagendrababu, Ove A. Peters, Amr Fawzy, Umer Daood

**Affiliations:** 1 Division of Clinical Dentistry, School of Dentistry, International Medical University Kuala Lumpur, 126, Jalan Jalil Perkasa 19, Bukit Jalil, Wilayah Persekutuan, 57000 Kuala Lumpur, Malaysia; 2 College of Dental Medicine, University of Sharjah, Sharjah, United Arab Emirates; 3 Department of Endodontics, Arthur A Dugoni School of Dentistry, University of the Pacific, San Francisco, CA USA; 4 School of Dentistry, University of Queensland, Herston, QLD 4006 Australia; 5 UWA Dental School, University of Western Australia, Nedlands, WA 6009 Australia; 6 Clinical Dentistry, Restorative Division, Faculty of Dentistry, International Medical University Kuala Lumpur, 126, Jalan Jalil Perkasa 19, Bukit Jalil, Wilayah Persekutuan, 57000 Kuala Lumpur, Malaysia

**Keywords:** Biophysics, Materials science

## Abstract

To evaluate structural profiles and mechanical behaviour of WaveOne Gold (WOG), Twisted File Adaptive (TFA) and XP-endo shaper (XPS) instruments after root canal preparation. Standardized in vitro shaping was performed in presence of 5.25% sodium hypochlorite. File morphology was analyzed using scanning electron microscopy; X-ray diffraction analysis was performed before and after use along with Raman spectroscopy. Nanoindentation was carried out to characterize surface topography. Ni^2+^ release was measured at 1, 3, 5 and 7 days. X-ray photoelectron spectroscopy (XPS) analysis was done before and after use. After allocating scan line shifts like in WOG, mechanical deformation was shown using first order polynomials. XPS file system showed minimal grooves on surface. SEM of WOG instrument showed scraping surface defects. Hardness varied from 8.11 ± 0.99 GPa in TFA system to 6.7 ± 1.27 GPa and 4.06 ± 4.1 GPa in XPS and WOG. Ni^2+^ concentration from WOG was 171.2 μg/L. Raman peak at 540–545 cm^−1^ is attributed to Cr_2_O_3_. High resolution of Ti 2p spectrum show distinctive peaks with binding energies dominating in WOG, XPS and TFA file system. XRD exhibited NiTi phases with diffraction peaks. WOG files showed more surface deterioration and less passive layer formation as compared to TFA and XPS systems.

## Introduction

Root canal preparation is a central step in endodontic therapy, which influences the success of the procedure. It involves removal of pulpal tissue and infected dentin, reducing microbial count, creating access for irrigants and intracanal medicament delivery and optimizing root canal system for root canal filling materials^1,2^.

Nickel-titanium (NiTi) instruments have become popular in endodontics because of their greater flexibility compared to stainless steel instruments, which results in fewer procedural errors^1,3^. However, the disadvantage of NiTi instrument is unexpected instrument fracture^4^, which compromises the root canal disinfection^5^. Various surface defects, such as pitting, metal flash and grinding marks on the instrument may act as stress concentration points, which reduces the resistance to fatigue fracture^6–8^.

In recent years, root canal instruments have been introduced in the market with lot of improvements in the instrument design, manufacturing process and kinematics to reduce the incidence of instrument fracture^4,9^. Thermal treatment is one of the approaches toward adjusting the transition temperature in NiTi alloy, which influences the flexibility and fatigue resistance of NiTi instruments^4^. Reciprocating motion is an innovation in nickel–titanium (NiTi) systems that claims to reduce the incidence of instrument fracture^10^.

WaveOne Gold (WOG) (Dentsply Maillefer, Ballaigues, Switzerland) is a single-file reciprocating system with parallelogram cross-sectional design, two cutting edges; the set consists of four instruments (20/07, 25/07, 35/06 and 45/05). WOG is manufactured using a specific thermal treatment, consisting of heating-annealing cycles applied after milling, in contrast to the premanufacturing heat treatment that is used for M-Wire production. The manufacturer claims that gold treatment technology will improve the strength and flexibility of the instrument^11^ (*WaveOne Gold, Surf the Canal with Confidence, Dentsply Sirona Endodontics*).

Twisted File Adaptive (TFA) (SybronEndo, Orange, CA) combines continuous and reciprocating motions. The TFA instrument detects torque within the motor (Elements Adaptive motor, SybronEndo) and changes the motion from continuous to reciprocating. In reciprocating motion, the instrument is rotated up to 370° clockwise and 50° counterclockwise directions. This adaptive technology is claimed to allow the instrument to advance into the root canal in clockwise direction and to reduce the risk of instrument fracture in counterclockwise directions. Additional features, like the so-called R-phase heat treatment, twisting of the metal for file production, an equilateral triangular cross section, and special surface conditioning are thought to potentially improve strength, flexibility, and fatigue resistance of TFA system (SybronEndo, Orange, CA). TFA files are marketed with reference to their use in different canal sizes. The Small Pack (one color band) and its instrument sequence (SM1 20/0.04, SM2 25/0.06) are used for small canals (SM); while the Medium/Large Pack (two color bands) and its instrument sequence (ML1 25/0.08, ML2 35/0.06) are used for medium or large canals (ML).

The XP-endo Shaper (XPS) (FKG Dentaire SA, La Chaux-de-Fonds, Switzerland) is a single file rotary system, manufactured from so-called MaxWire nickel-titanium alloy. The instrument body itself has 1% taper in martensitic phase at room temperature; when submitted to body temperature, the instrument assumes a characteristic s-shaped appearance and will develop, depending on the initial canal shape and the dwell time in the canal, a taper of about 4%. The tip design of XPS has six cutting edges and a smooth transition from the base of the tip to the helical shaft known as a “booster tip”. The manufacturer claims that its design and the alloy used make the file very flexible, which reduces the stress in root canal walls and improves cyclic fatigue resistance.

A clinician must understand the nature of different NiTi manufacturing process and kinematics, to select the appropriate instrument to achieve successful root canal treatment. Although manufacturers of almost all single file systems recommend disposing of the instrument set after one clinical use, there is a sizeable anatomical variation amongst different teeth with low fracture incidence indicating safe use of multiple preparations of root canals after strictly following manufacturer’s instructions^12^. A possible clinical implication of such study is that the risk of instrument fracture cannot be gauged or understood safely based on visual assessment for the degree of wear of its external surface. Research is important in order to identify what combination of these factors promoting greater safety and efficiency, based on the progression of its deterioration during use. To the best of the authors’ knowledge, so far no study has directly compared the surface properties, chemical composition and mechanical characteristics among various manufacturing processes (Gold treatment, R-phase heat treatment and MaxWire nickel–titanium alloy) combined with different kinematics (rotary, reciprocation and adaptive motion). Scanning electron microscopy (SEM) has proven itself to be one of the most effective microscopy methods offering ease of use and high spatial resolution revealing in-plane geometrical properties in experimental micromechanics^13^. The mapping function is parametrized by a set of degrees of freedom (dofs) helping in achieving different approximations for image gradients^14^. The use of analytical techniques can provide important additional information for the endodontic instruments after use. The results are presented and discussed with respect to the material parameters for structural transformation and chemical composition using X-ray Diffraction analysis, X-ray photoelectron spectroscopy (XPS) and Raman spectroscopy. To establish the composition and microstructure analysis relationship after use, the mechanical properties have been studied by application of nanoindentation to investigate deformation^15^. Due to its displacement sensing and precise load controlling, nanoindentation has been used to study mechanical response of materials such as hardness^16^. Our study also aims at exploring the atomic scale Ni^2+^ release study to provide a scientific basis of surface analysis. Therefore, the current in vitro study aimed at three objectives: (1) to evaluate and compare the chemical analysis for structural profiles of WOG, TFA and XPS after root canal preparation using Raman spectroscopy, (2) to evaluate and compare the surface analysis of WOG, TFA and XPS after root canal preparation using scanning electron microscope (SEM) and X-ray diffraction (XRD) analysis, (3) to evaluate and compare the mechanical behavior analysis of WOG, TFA and XPS after root canal preparation using nanoindentation analysis. The null hypotheses tested were that the structural profiles and mechanical behaviour of WOG, TFA and XPS instruments will not be significantly altered after root canal preparation.

## Results

**Figure 1:**
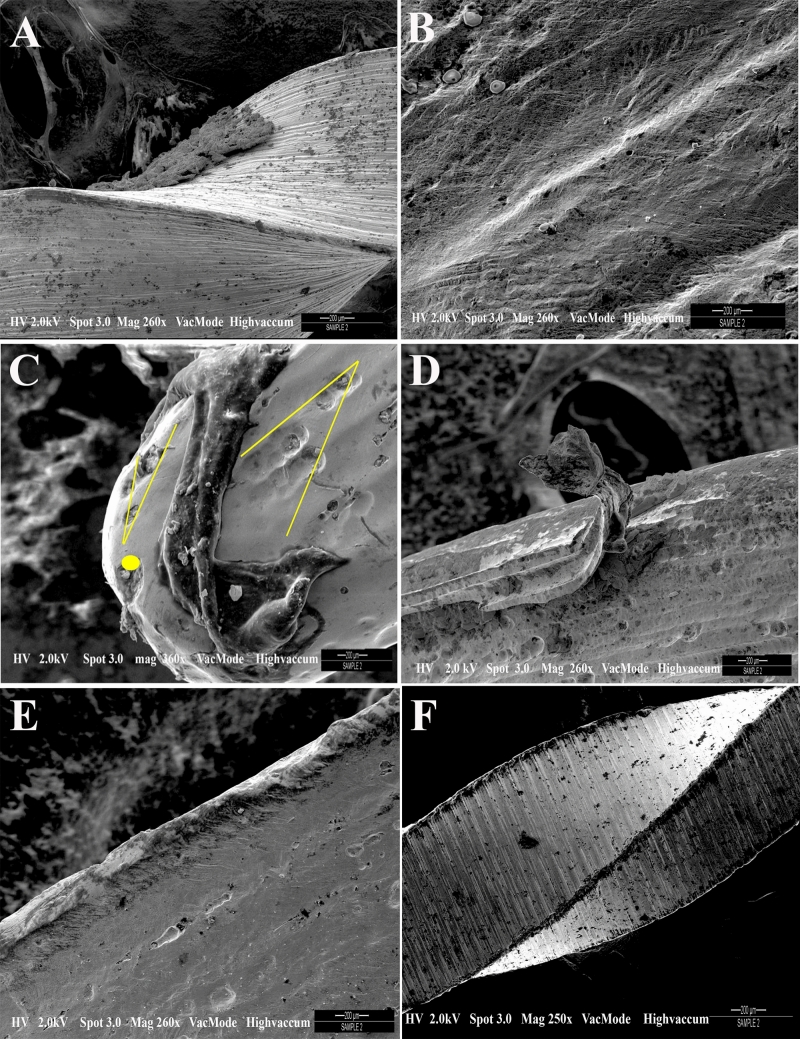
SEM micrographs of three different endodontic instrument systems showing different surface characteristics. (**A**) XPS file system showing the presence of minimal grooves on the instrument surface; (**B**) TFA file system showing absence of cracks or grooves on the surface of file system; (**C**) SEM micrograph of the final 3 mm of WOG instrument showing the transition angle of surface defects on instrument surface. The yellow lines are depicting the process of correcting scan line shift artifacts in SEM images (**D**) SEM micrograph of WOG system showing metal strips and scraping on the instrument surface (**E**) compared to the XPS system after one use (**F**) Image of XPS file system after one use.

The SEM micrographs of the three endodontic instrument systems show different surface characteristics (Fig. 1). The scan line shifts had an amplitude ranging up to 5 pixels. These scan line shifts were formed due to the positioning errors of the electron beam. Next, the same images were corrected for the observed scan line shifts. After allocation scan line shifts like in WOG, the mechanical deformation is shown using first order polynomials. This enabled a precise detection of the scan line showing a mechanical artifact and a defect. The proposed procedure is applied to the three images (Fig. 1A–C). The XPS file in Fig. 1A is showing the presence of minimal grooves on the instrument surface. SEM micrograph of the final 3 mm of WOG instrument showed the transition angle of surface defects on instrument surface (Fig. 1B). The scores for the type of defects at the tip and 10 mm down are shown in Table 1. The TFA at a higher magnification showed absence of cracks or grooves on the surface of file system (Fig. 1B). The files showed lesser defects such as microcavities and irregularities. The number of grooves, irregularities and microcavities were seen in the files from WOG as compared to the other groups. SEM micrograph of WOG Primary (Fig. 1D) showed extruded and scraping on the instrument surface as compared to the XPS system after one use.

**Table 1:** Presence of the type of defects of the groups Twisted File, Wave One Gold and XP Endo shaper.

Groups	Irregular		Grooves		Microcavities	
	Tip	10 mm	Tip	10 mm	Tip	10 mm
Wave One Gold	9	4	6	7	7	8
Twisted Apical File	4	1	1	0	3	2
Endo Shaper	1	1	2	1	5	2

**Figure 2:**
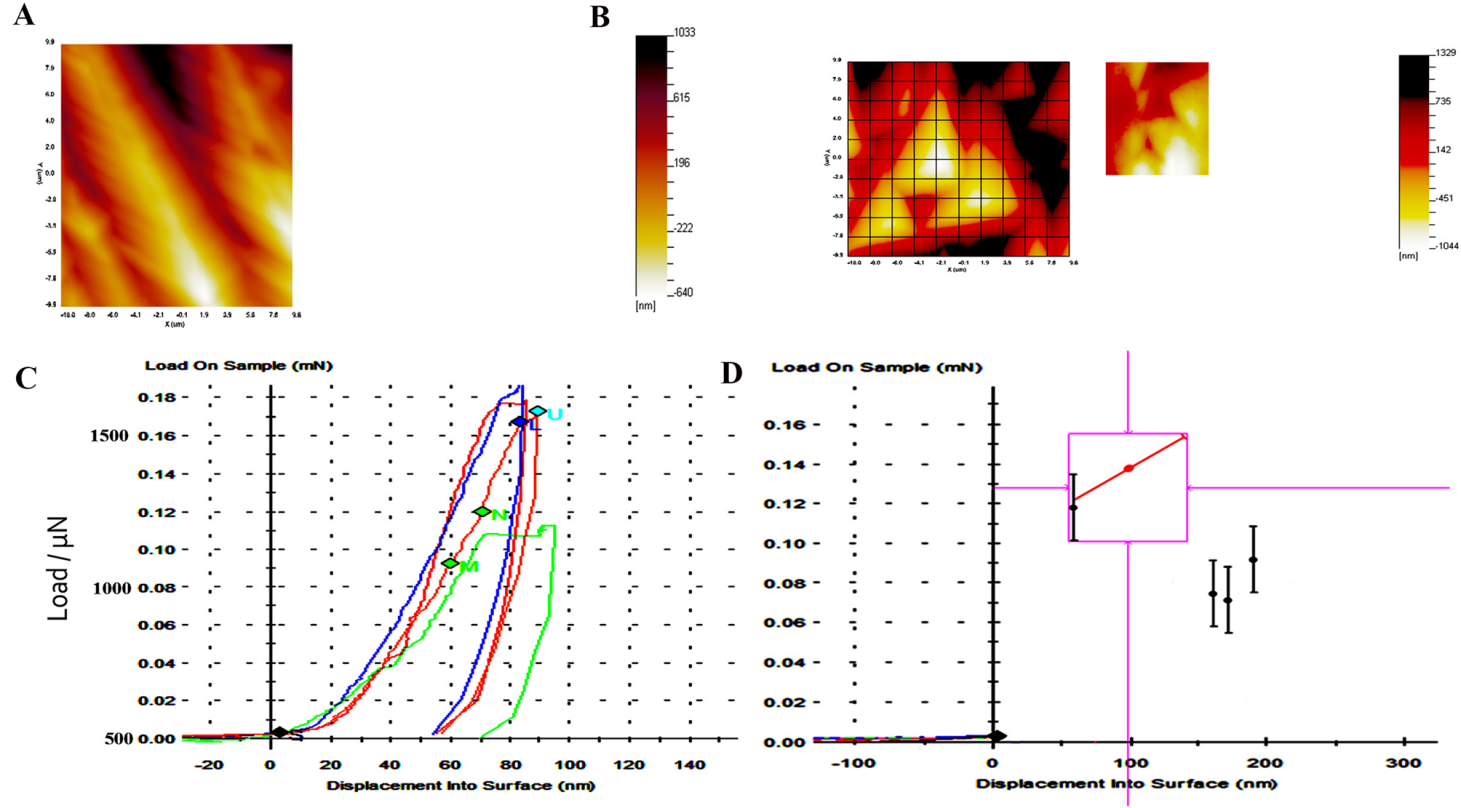
(**A**, **B**) corresponding AFM image (inset plan view optical microscope image at × 40 magnification) at mN load (**C**) Load–displacement curves for nanoindentation of all specimens numbered, blue > control TFA file, red > TFA instrumented file, red/U > XPS file, green > WOG, with (**D**) individual hardness vs. displacement curves.

There are differences in values appearing in indent amongst all systems (Fig. 2A, B), and slope of nanoindentation unloading part determined hardness varying from 8.11 ± 0.99 GPa in TFA system to 6.7 ± 1.27 GPa and 4.06 ± 4.1 GPa in XPS and WOG respectively (Table 2). From the figure (Fig. 2), there is divergence in the slope of unloading and maximum indent depth determining the hardness of different file systems (Fig. 2C). Table 2 lists the measured nanoindentation values. It is noticed that the nanohardness values exhibit the similar trend as the N^2+^ release pattern amongst different systems. The WOG, TFA, XPS file exhibited the hardness values 4.06 ± 4.1, 8.11 ± 0.99 and 6.7 ± 1.27, respectively. It is worth mentioning that in this study, the hardness of all file systems is considered higher.

**Table 2:** Means ± standard deviations of the variations in the surface mechanical properties (nano-indentation) in terms of hardness (H); Ni^2+^ release rate and concentration in the medium solution for samples as a function of the immersion time kept at 37 °C.

Groups	1st day	3rd day	5th day	7th day	*p*
Wave one gold protocol (WOG)	171.2 μg/L ± 23.6	179.3 μg/L ± 19.1	154.1 μg/L ± 29.9	121.5 μg/L ± 14.5	*p* < 0.05
	4.06 ± 4.1 (H)				
Twisted Adaptive File	47.4 μg/L ± 9.9	39.5 μg/L ± 6.7	31.6 μg/L ± 9.1	27.9 μg/L ± 7.7	*p* < 0.05
	8.11 ± 0.99 (H)				
XP Endo Shaper	69.3 μg/L ± 12.1	58.7 μg/L ± 15.1	49.1 μg/L ± 12.1	39.5 μg/L ± 6.3	*p* < 0.05
	6.7 ± 1.27 (H)				

**Figure 3:**
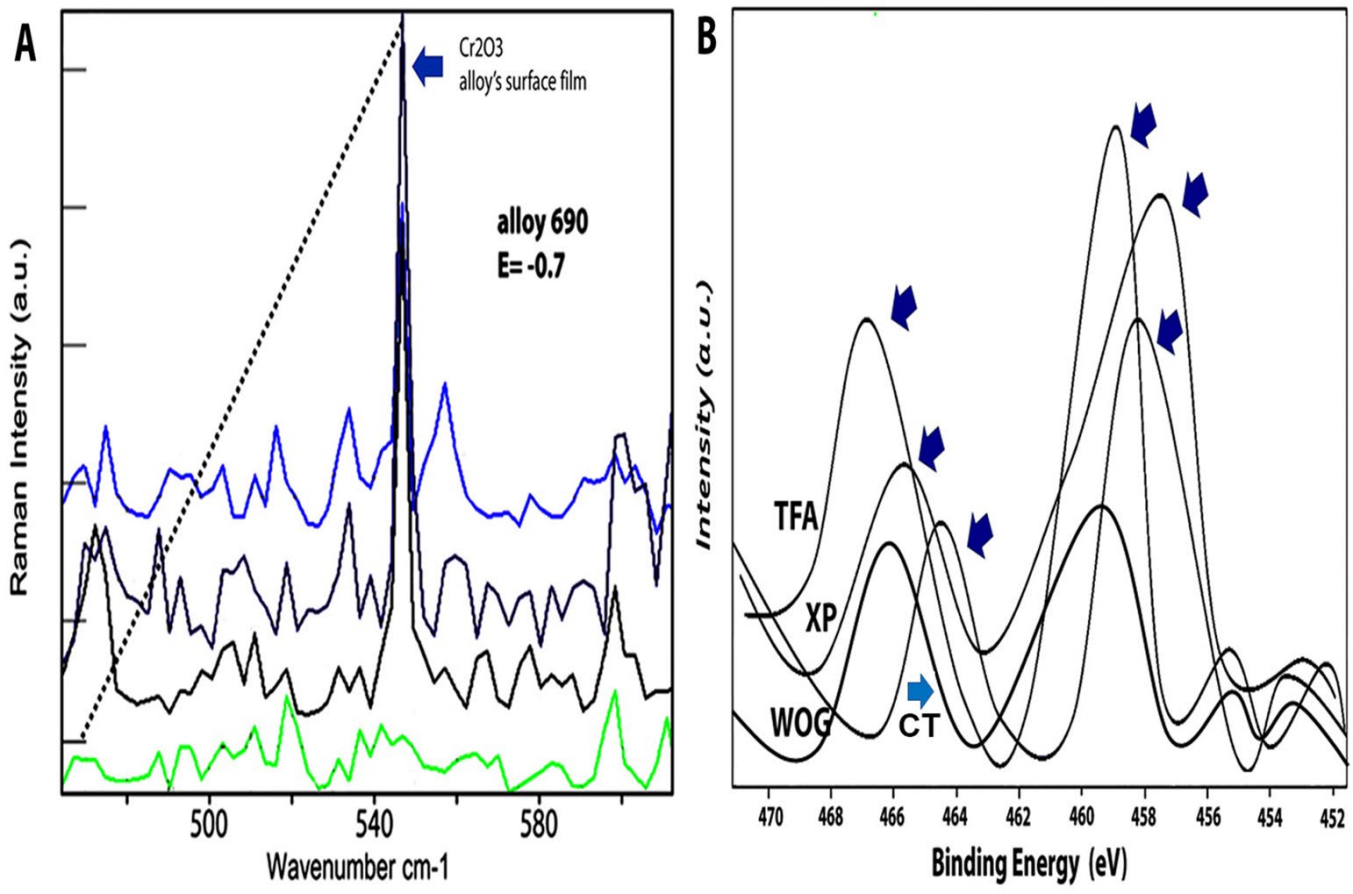
(**A**) Raman spectrum of specimens for prominent band with Raman peak located at 540 cm^−1^. An analysis of Raman spectra tabulated indicate alloy’s chromium concentration increases, the amount of Cr_2_O_3_ included in the alloy’s surface film would increase. The intensity is highest found in TFA files (light blue spectrum) > followed by XPS Endo system (dark blue spectrum) > followed by Wave One Gold system (black spectrum) as compared to control spectrum (green); (**B**) XPS spectra of the three file systems (TFA, WOG, XPS) and control (CT) used in the region of Ti 2p, and Ni 2p.

The Raman peak at 540–545 cm^−1^ is attributed to the same position as the most intense Raman peak for Cr_2_O_3_. Clearly in the Raman spectrum, the results strongly suggest that the amount of Cr2O3 formed increases in the alloy’s chromium concentration in TFA system (Fig. 3A). From the general observation that the protectiveness of a surface oxide is inversely proportional to the WOG system used which showed the least intensity. Thus, it might be the case that the corrosion resistance of the file system increases with the alloy’s chromium concentration. The XPS spectra in the regions of Ti, and Ni of the file systems are represented with high-resolution spectrum for Ti and Ni showing distinctive binding energies of 464.1, 466, and 467.6 eV dominating in WOG, XPS and TFA file system respectively (Fig. 3B). The Ti contributions also decrease simultaneously in the same order of the file systems.

**Figure 4:**
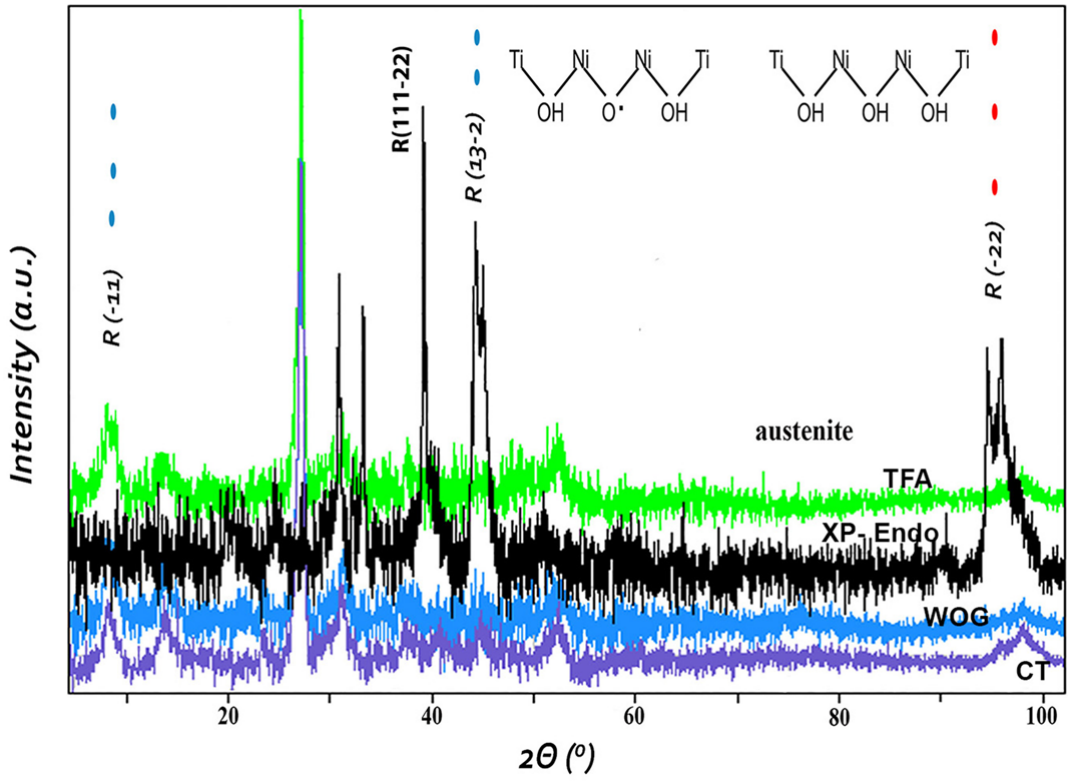
XRD patterns of the tested files after one use. Purple—control unused wave one gold for standard; blue—WaveOne gold (WOG)—black—XP-endo shaper(XPS); green Twisted File Adapted (TFA) after use.

The intensity of XRD peaks increased intensity with the formation of Cr_2_O_3_ exhibiting presence of NiTi phases with diffraction peaks at 40, 47 and 53. The analysis software also confirmed the content of NiTi at a higher intensity for TFA as compared to the least intensity found in WOG. The sharp XRD peaks between a 2θ range of 28–38 confirms the crystalline phase seen at highest intensity for TFA file system giving a better idea of corrosion resistance of the used file system (Fig. 4). The broad contribution location of 42.12° for different file systems used with highest intensity for TFA system represents the plane of the austenitic phase of the NiTi system with no shifts but change in intensities.

After 24 h immersion in a salt solution, Ni^2+^ concentration released from WOG was 171.2 μg/L, whereas that for TFA and XPS was 47.4 μg/L ± 9.9 and 69.3 μg/L ± 20.1, respectively. After 7 days, a plateau was reached as the released concentration of Ni^2+^ had decreased to 121.5 μg/L ± 14.5 for Wave One Gold, which was still significantly higher compared to 39.5 μg/L ± 6.3 for XP Endo shaper (*p* < 0.05). The results demonstrated that WOG had more Ni^2+^ release as compared to the other file systems used. The TFA and XPS were significantly different to WOG and amongst as the values were proportionately significant on each day from 1st till 7th day (Table 2).

## Discussion

The design and the materials used in endodontic files have several variations, which have great influence on the properties of the file system. It is imperative for the clinicians to know and acknowledge the properties of the different file systems used and the differences of the materials used in new technologies^17^. The current in vitro study has compared the mechanical and surface change properties between three systems during canal instrumentation. There are significant factors that affect the properties, and chemical composition of the alloy^18–20^. Endodontic treatment has made great progress with the inclusion of NiTi instruments, but risk of fracture is always there due to various causes^21^. Many intrinsic factors of the instrument, such as cross-sectional dimension, size, taper, and alloy composition, along with canal anatomy can affect different mechanical properties^22^.

Let us consider the scan line as exhibited as a clear scan line shift in the result for the SEM images. These are formed as a result of distinct jumps within the displacement field which are either positive or negative occurring in the scanning direction. These originate from the SEM imaging processes^23^. The scan line seen in the SEM Fig. 1C represent the actual positions where intensities are registered typically having scan line spacings. This was done after performing a pre-correlation between the images. The use of instruments for endodontic procedures produces surface deterioration after instrumentation, which indicates a need of instruments that would stand up to more than single-tooth use, despite the fact there is only one-use recommendation by the manufacturers. The scores for the type of defects at the tip and 10 mm down indicate irregularities, grooves and microcavities. There is an important detail, which is reduced defects seen in TFA and XPS files. In increasing order of defects, WOG showed more microcavities, grooves and irregularities, whereas TFA system showed least number of microcavities. These findings were confirmed in where TFA file system showed absence of cracks or grooves on the surface of file system in most places. As for the findings, it must be considered that there was an improvement in the production methods^24^. There has been an improvement in the nickel–titanium rotary instruments of different brand systems regarding instrument wear^25^, our observation was they did not deform as expected except the WOG. The instruments are used without first preliminary coronal enlargement resulting in greater engagement of the file flutes producing more torque and/or applied pressure on the file system. The WOG files were introduced as an update to the WaveOne (WO, Dentsply Maillefer) with heat-treatment procedure for gold alloy resulting in increased flexibility as well as cyclic fatigue resistance^26^. The manufacturer claims that with this new heat treatment, there is improvement in elasticity and cyclic fatigue resistance of the file system (WO, Dentsply Maillefer). It is noteworthy that the TFA file has a production method which is different from the rest of the systems as the active part is made by twisting^27^ however, the file system showed lesser defects and had no negative influence on the surface which was contrary to previous studies^28^. Perhaps this system is more resistant to changes due to its new torsional fabrication process^29^. For XP Shaper, the manufacturer has stated that the technology design provides superelasticity and extreme flexibility^30^. Elnaghy claimed highest cyclic fatigue resistance between the tested instruments, which is consistent with lesser defects in our study, showing some form of resistance. The presence of MaxWire alloy makes it super elastic having shape memory^30^. Moreover, the WOG file system showed the least values, which was in line with the SEM experimental analysis (Fig. 2). Previously, the dynamic cyclic fatigue resistance was found to be less for WOG systems when compared to other file systems (for example Reciproc Blue) and dynamic cyclic fatigue resistance was significantly higher than the static cyclic fatigue resistance^31^. Conversely, Mehmet et al. indicated that WOG instruments were more resistant to cyclic fatigue^32^.

After 24 h, it was evident that WOG Ni^2+^ release concentration was the highest (171.2 μg/L ± 23.6) which went up to the 7th day; whereas that for the TFA and XPS, values were far lesser (Table 2). The authors speculate that there is an effective protected layer for inhibiting the Ni^2+^ leaking. The best way of distinguishing the absence or presence of the Cr_2_O_3_ was the presence of peak at 540–560 cm^−1^ region (Fig. 3A). The spectra obtained from the surface of TFA and XPS exhibited a fairly high intensity at the region, while the other set of spectra for WOG exhibited a relatively weaker peak. Distinguishing the Cr_2_O_3_ is particularly challenging because the Raman peaks are very accurately located at 540–560 cm^−1^ region and is symmetry forbidden^33^. Generally, a Raman peak at 610 cm^−1^ is also indicative of Cr_2_O_3_, but the peak was not visible in either of the file system. Therefore, even in the absence of a peak at 610 cm^−1^, the Raman peak at around 540 cm^−1^ regions was best assigned to the Cr_2_O_3_ layer. The presence of Ni^2+^ concentration which was not released in greater quantities in TFA and XPS s, indicates an increase in the oxygen ion concentration. This maintains a charge neutrality increasing the concentration of oxidation^33^ and integrated intensities at 540 cm^−1^ was a function of the file’s chromium protection.

The XPS spectra in the regions of Ti, and Ni of the file systems are represented with high-resolution spectrum for Ti and Ni showing distinctive binding energies of 464.1, 466, and 467.6 eV dominating in WOG, XPS and TFA file system respectively. The Ti contributions also decrease simultaneously in the same order of the file systems. The as-received XPS analysis proves the presence of TiO_2_. This rise of intensity may have been due to the grainy surface of the XPS and TFA systems because of TiO_2_. Also, within the XRD patterns, the peaks obtained are presented in Fig. 4. The patterns presented the NiTi phases and the sharp XRD peaks between a 2θ range of 28–38 confirms the crystalline phase seen at highest intensity for TFA file system giving a better idea of corrosion resistance of the used file system (Fig. 4). The peaks around 2θ range of 40–47 were the ones related to the austenite phase with 2θ range of 28 confirming the martensitic phase. The peaks showed variable intensity, with highest peaks seen in TFA files as compared to the WOG which showed the lowest intensities. The only difference between the other files was the intensity of the austenitic peaks. The intensity of austenite peaks in TFA system was lesser as compared to other files. This was a clear indication that the file system contained more martensitic phase. Intermetallic phases were prevalent in the TFA and XPS systems, which are homogenous phases and can affect mechanical and shape memory properties^34^. The TFA system uses adaptive motion that adjusts to instrumentation stress process automatically. The system has an interrupted continuous rotation which allows optimum cutting efficiency with considerable removal of debris adaptive movement and reciprocation, which are meant to improve the cyclic fatigue without affecting performance^35^.

The tip sizes and tapers of WOG, XPS and TFA file systems are different. WOG file with its parallelogram cross-sectional design and only two cutting edges decreases the torsional stress on the file^36^. However, TFA file has a triangular cross-section design having comparable cyclic fatigue resistance coupled with the novel adaptive motion operational mode^37^. All file systems differ in properties, geometry, and the mechanism of action. Collectively, the data favors the rejection of null hypothesis as the structural profile and mechanical behavior of three included instruments were altered. Moreover, the surface grooves or pits in the root canal instrument might act as stress concentration points, which eventually reduce the resistance to fracture. Hence, the current study provides information about the mechanical and surface change of instruments to the clinician, which could help them in selecting most appropriate instruments and discard the deformed used instruments on a rationale base. In addition, a level of difficulty was included using only molar teeth because of the presence of curved canals^38^. However, while this conclusion may be revealing and relevant, it is based entirely on empirical in vitro observation, and not on the concrete clinical data observed during the progressive deterioration process of the instrument, ultimately associated with clinical implications. However, the outcomes could be relevant because they provide clinicians with an indication of possible clinical applications and limitations of these files. In addition, other limitations included a difficulty of analyzing the same instruments at different time points of the research. Hence the specimen analysis precluded other use of instruments. Moreover, type of teeth and root canal, morphology of the root canal (curvature) were standardized and to reduce the operator variability, single operator performed the root canal instrumentation for all the groups, suggestive of viable strengths within the study. In any event, all the analytical techniques performed, lend themselves to the accurate evaluation of endodontic file surfaces.

## Conclusion

Available evidence is limited, but WOG system showed more surface deterioration and less passive layer formation as compared to TFA and XPS systems.

## Materials and methods

The protocol of the study design was approved by International Medical University Joint-Committee on Research and Ethics (IMU 429/2019). Patients who had donated extracted teeth were informed for consent and were provided enough information in a way they can understand (all subjects were above 18 years). The informed consent was obtained from all subjects. All methods were carried out in accordance with relevant guidelines and regulations. The rotary instruments investigated assessed in this study (*n* = 20) were WaveOne gold Primary (WOG) (Dentsply Sirona, Ballaigues, Switzerland), Twisted File Adaptive (TFA) (SybronEndo, Orange, CA, USA) files and XP Endo (XPS) (FKG Switzerland) files. Before root canal instrumentation, each instrument had been inspected under the microscope (Zeiss Stemi 2000-C, Carl Zeiss Jena GmbH, Zeiss Group, Jena, Germany) to identify any defects in the instruments. The instrument with presence of cracks/irregularities were to be excluded from the study. The sample size for analysis of instruments are derived (α = 0.01; β = 0.20; σ = 20.0; δ = 20.0) keeping the power of study equal to 90% and level of significance equal to 5% (Marco et al. 2019).

A total of 60 human maxillary first molars were collected and stored in 1% thymol solution until further use. Teeth with open apices, fractures, previous root canal-filled, calcified root canals and internal/external root resorption were excluded. Mesiobuccal roots with 10°–20° curvature determined according to the Schneider method (Schneider 1971) were included in the current study. The teeth were sectioned at cementoenamel junction using diamond disc and the canal orifices located. Patency of mesio buccal canal (not deploying MB2) was confirmed using a #10 or 15K file (SybronEndo); working lengths (WLs) were established by placing a #10K-file so that it was just visible past the apical foramen and reducing the length by 1 mm. Any root canal bigger than initial apical file size #20 was excluded from the study. Specimens were randomly allocated to three experimental groups (n = 20) according to the system used for the root canal preparation: WOG, XPS (VDW GmbH gold Reciproc motor; Munich, Germany), TFA and TFA (Elements Adaptive motor, Sybron Endo; Kerr Dental CA, USA). The speed and torque used was based on manufacturer’s instructions. After a glide path was created canals were instrumented with rotary instruments following manufacturers’ instructions. Each instrument is used to prepare only one canal.

### Root canal instrumentation

#### WaveOne Gold

The shaping was performed using the primary #25/0.07 instrument in the presence of 5.25% sodium hypochlorite. Gentle inward pressure was used to let the primary file go passively. After shaping 2–3 mm of the given canal, the primary file was removed, cleaned, and then the canal was irrigated, recapitulated with a size #10K-file followed by re-irrigation. Continuing with the WOG primary, in 2–3 passes, the coronal two thirds of the canal was enlarged, a brushing motion on the outstroke to eliminate coronal interferences was utilized. The file was then carried to WL in one or more passes. Upon reaching the working length, the file was removed to avoid over-enlarging the foramen.

#### Twisted File Adaptive

The green instrument was slowly advanced (SM1 (20/0.04)) with a single controlled motion until the file engaged inside the dentin; later the file was completely withdrawn from inside the canal. During the entire process, the file was not forced apically. The flutes were wiped off and NaOCl delivered to the pulp chamber, and patency confirmed using a size #15K-file, repeating the process with a yellow instrument (SM2 (25/0.06) until that file reached WL.

#### XP-Endo Shaper

Once a glide path was confirmed, shaping was initiated using XP-endo shaper with gentle strokes to progress to the WL. If WL was not achieved in five strokes, instrumentation was stopped, and irrigation was done with recapitulation and the process started again. Any pecking motion was avoided with the instrument always in spinning movement while inside the canal. Once WL was reached, the canals were irrigated, and the instruments were worked for 15 additional long gentle strokes to perform enlargement at WL.

Each set of root canal instruments was used only in one canal and then the instrument has been discarded. All instruments were used until WL and canal patency was rechecked using size 10K-file. For all the three groups, root canal was irrigated with 5.25% sodium hypochlorite delivered by 30-gauge side vented needle (Diadent, Korea) after use of each instrument with a total of 20 mL per canal. *WOG—*25/0.07, TFA—SM2 25/0.06, XPS—30/0.04 was used for analysis. To reduce operator variability, instrumentation was performed by a single operator (SHB) trained in all the three root canal instrumentation techniques.

### Scanning electron microscopy

After root canal instrumentation, the morphology and surface analysis of the instruments were analyzed using scanning electron microscopy (SEM). A morphological characterization was performed by Field Emission-SEM technique (FEI STRATA DB 235M, Hillsboro, OR, USA) using a field emission gun with 2 nm resolution (*n* = *3/group*). Local corrosion points were investigated using focused ion beam (Ga+ ions). The samples were tilted at 55° to evaluate the microstructure changes in the exact position within *situ* platinum deposition. In order to detect any possible scan line shifts in the images, a precorrelation between the images were performed using a set of polynomials, i.e. six degrees of freedoms (dofs).

The dofs represent the mapping functions, both mechanical and imaging-related as each mapping function relied on the degrees of freedom. The polynomial was matched with the displacements defining the deformation phase in an average. The allocation of line shifts was performed between the selected images (*n* = *3/group*) of each specimen, with three separate pre-correlations on three images of the same area of each specimen. The y-coordinate of each row determined the horizontal axis representing a scan line shift existing in the image using an algorithm artifact mapping function assigning *ɸ*_*f*_ and *ɸ*_*g*_ for a final correlation within ± 15 pixels. Each analysis was performed in two steps with amplitudes of x and y directions and position of scan lines considered as degrees of freedom.

Several speckle pattern images (*n* = *3/group*) were produced with known distortions and scan lines computed at different magnifications. Images were also examined by three independent and blinded evaluators for alterations and defects of the files; their criteria for such evaluation were the presence of irregular edges, grooves, and microcavities in comparison to the control specimens (unused instruments).

### X-ray diffraction analysis

The X-ray diffraction (XRD) analysis of specimens (*n* = *3*/group) before and after instrumentation was performed using X-ray diffractometer (Rigaku, Japan) operating at 40 kV/30 mA. Each specimen was performed at 1.5406 (λ) wavelength. The pattern was recorded between 20 to 80° using a step size of 0.02°. The microstructure of samples was also observed using an x-ray diffractometer Siemens d5000, Siemens, Munich, Germany).

### Raman data acquisition

Raman spectroscopy measurements were performed using a Leica microscope and lenses (JY LabRam HR 800, Horiba Jobin Yvon, France) with curve-fitting Raman software (Labspec 5) using a monochromatic green laser (532 nm, 8 mW incident power, ThorLabs, Newton Avenue, New Jersey, USA). This was alongside a long pass filter and a beam splitter (Razor Edge, Semrock). A CCD camera (IXON, Andor, Oxford Instruments) with a spectrograph (SP-2300i, Teledyne Princeton Instruments) was used to collect data over an exposure time of 1 s. An average of 10 measurements were taken at 50 × magnification and a spectral resolution of 1 cm^−1^ (natural line widths of Raman lines) and a laser power of 4.94mW recorded from different locations. Readings were taken from the central region of the files (*n* = *5*) ending onto the left side of the specimen in 1 µm steps using the *x–y–z* stage with background-corrected counts.

### Nanoindentation analysis

To make sure orientation and hardness relationship was maintained, indentations (*n* = *3/group*) were carefully selected in the middle of the specimen files identified by Electron backscatter diffraction (EBSD) mapping using ZEISS 55 SEM. Nanoindentation was carried out using Hysitron nanoindenter (TI-950, Hysitron Inc., USA) with a Berkovich indenter (3400, Tucson AZ, 85706, USA). The deformation of one indentor did not interfere with another indentation deformation making the spacing 20 times for the maximum width. Loading procedure was as follows; maximum load of 2000 µN for 5 s, holding for 2 s and then unloading to 0 µN for another 5 s under a constant strain via standard continuous stiffness measurements varying between 0.2, 0.09 and 0.01 s^−1^. This was repeated using four repeated indents with peak load set at 10 mN and preload P_0_ set as 500 µN. Nanoscope Analysis software in Bruker Dimensions Icon Atomic Force Microscopy (5848 Microtester, Instron, Canton, MA, USA) were used at room temperature for surface topography around the indents.

### Nickel ion release characterization

The specimens (*n* = *3/group*) were partially covered with lacquer considered impermeable to ions defined measuring fields and edge effects were eliminated. To verify the release of Ni^2+^ ions, files were spot fixed at 37 °C in silicon rubber tubing with capacitance of 4 nF and capacity of 10 mL. The tube was filled with 10 mL NaCl solution at a concentration of 0.9% and Ni^2+^ release was measured at 1, 3, 5 and 7 days by means of Inductively Coupled Plasma Mass Spectrometry (ICP-MS) method at room temperature having electrostatic lenses that focus (positive) ions onto the entry to the mass-spectrometer (AB Sciex Pte. Ltd, Central Indus Estate, Singapore).

### X-ray photoelectron spectroscopy (XPS) analysis

The XPS analysis before and after use of the file (*n* = *3*/group) and Ni/Ti control was performed using Axis-NOVA (Kratos, UK) with a monochromatic Al Kα X-ray source (1486.6 eV) and an aluminum mode. A pass energy of 20 eV with energy steps of 0.2 eV step^−1^ was maintained for the photoelectron spectra. The photoelectron take-off angle was 90° between the sample surface and the axis of the analyzer lens with the aperture of spectrometer set at 300 × 700 µm^2^. Charging effect compensation was done using the magnetic immersion lens of a flood gun, in the XPS instrument. Depth profiling was done using Ar ions at 3000 eV in each 10 s cycles. Data was fit using Smart peak ground parameters in Avantage 5.9 software. Calibration was done by setting the C 1 s signal peak from impurities (284.8 eV.).

### Statistical analysis

Data were expressed as means ± standard deviations (*n* = *3*/group) and analyzed by two-way ANOVA followed by Tukey’s test for repeated-measurement pair-wise comparison within the time points for Ni^2+^ release. The remaining data and standard deviations were analyzed using one-way ANOVA. A level of *p* < 0.05 was considered statistically significant.
