# Author Correction: Evaluation of usage-induced degradation of different endodontic file systems

**DOI:** 10.1038/s41598-021-94982-z

**Published:** 2021-08-02

**Authors:** Shekhar Bhatia, Venkateshbabu Nagendrababu, Ove A. Peters, Amr Fawzy, Umer Daood

**Affiliations:** 1 Division of Clinical Dentistry, School of Dentistry, International Medical University Kuala Lumpur, 126, Jalan Jalil Perkasa 19, Bukit Jalil, Wilayah Persekutuan, 57000 Kuala Lumpur, Malaysia; 2 College of Dental Medicine, University of Sharjah, Sharjah, United Arab Emirates; 3 Department of Endodontics, Arthur A Dugoni School of Dentistry, University of the Pacific, San Francisco, CA USA; 4 School of Dentistry, University of Queensland, Herston, QLD 4006 Australia; 5 UWA Dental School, University of Western Australia, Nedlands, WA 6009 Australia; 6 Clinical Dentistry, Restorative Division, Faculty of Dentistry, International Medical University Kuala Lumpur, 126, Jalan Jalil Perkasa 19, Bukit Jalil, Wilayah Persekutuan, 57000 Kuala Lumpur, Malaysia

Correction to: *Scientific Reports* 10.1038/s41598-021-88570-4, published online 27 April 2021

The original version of this Article contained an error in the spelling of the author Venkateshbabu Nagendrababu, which was incorrectly given as Venkatesh Nagendrababu. The original Article has been corrected.

